# Effects of fructo-oligosaccharides on genitourinary tract infections and birth outcomes in pregnant women: a randomized controlled trial in Bangladesh

**DOI:** 10.1186/s41182-025-00788-4

**Published:** 2025-08-13

**Authors:** Shamima Sultana, Yukiko Wagatsuma, Rumana Sharmin, Dilruba Ahmed, Arif Hasan Chowdhury, Ahshanul Haque, Tahmeed Ahmed, Harald Brüssow, Shafiqul Alam Sarker

**Affiliations:** 1 International Centre for Diarrhoeal Disease Research, Bangladesh (icddr,b), 68 Shaheed Tajuddin Ahmed Sharani, Mohakhali, Dhaka 1212 Bangladesh; 2 Institute of Medicine, University of Tsukuba, 1-1-1 Tennodai, Tsukuba, 305-8575 Japan; 3 Nestlé Research Center, Nestec Ltd, Vers-Chez-Les-Blanc, CH-1000 Lausanne 26, Switzerland

**Keywords:** Prebiotic, Fructo-oligosaccharide, Low birth weight, *Lactobacillus*, *Bifidobacterium*, Pregnant women, Genitourinary tract infections

## Abstract

**Background:**

Genitourinary tract infections, including bacterial vaginosis, which is characterized by the loss of *Lactobacillus* (LAB) in the vaginal microbiota, is a risk factor for low birth weight. The aim of this study was to examine the effects of fructo-oligosaccharide (FOS) supplementation on the incidence of genitourinary tract infections in pregnant women and the birth weights of newborns in Bangladesh.

**Methods:**

A randomized, double-blinded, placebo-controlled study was conducted in Dhaka, Bangladesh. Women in early pregnancy were randomized to the FOS or placebo groups (105 women per group), and supplements were provided daily until delivery. Stool samples were collected from women at baseline and at 24 and 36 weeks of gestation and from infants at birth for the analysis of LAB and *Bifidobacterium* by PCR. Vaginal swabs to test for bacterial vaginosis were collected at 18 and 30 weeks of gestation. Anthropometric measurements were taken at birth, and the newborns were followed up for 6 months.

**Results:**

Of the 210 pregnant women, 8 had abortions, 5 refused the study product, 31 migrated, 4 had infants who were stillborn, and the neonate of 1 woman died early. The mean (SD) birth weight was 2799 (381) grams; 27 (17.0%) newborns had low birth weight (15.6% in the FOS group and 19.5% in the placebo group). Birth weight did not differ between the groups after adjusting for gestational week at birth and maternal early pregnancy BMI. Bacterial vaginoses were observed in 4.3% of women in the FOS group and 3.1% of women in the placebo group and were not statistically different between the groups. LAB colonization rates in stools of pregnant women at 24 and 36 gestational weeks did not differ between the groups. However, LAB colonization rate was higher in stools of infants in the FOS group than in those in the placebo group (68.8% in the FOS group and 51.2% in the placebo group, *p* = 0.024). This difference remained significant after adjusting for maternal age and LAB colonization at baseline (adjusted risk ratio (95% CI) = 1.45 (1.12–1.88), *p* = 0.005). The rate of *Bifidobacterium* colonization in the stools of infants did not differ between the groups.

**Conclusions:**

FOS supplementation did not affect bacterial vaginosis incidence in pregnant women or infant birth weight. A higher rate of *Lactobacillus* in the stool samples of infants whose mothers received FOS was observed. Further studies are needed to confirm these findings with a large sample size.

**Trial registration:**

This study was registered at Clinicaltrials.gov (NCT02127225).

**Supplementary Information:**

The online version contains supplementary material available at 10.1186/s41182-025-00788-4.

## Background

More than 20 million infants worldwide, representing 16% of all births, are born with low birth weight (LBW), and 96% of them live in developing countries. LBW deliveries account for 40–50% of neonatal deaths [1, 2]. Moreover, for survivors of LBW, cognitive impairment and adult onset of chronic diseases have been reported [3]. Genitourinary tract infections (GUTIs), including bacterial vaginosis (BV), are reported to be important risk factors for LBW in both industrialized and developing countries [4–7]. GUTIs are associated with the loss of *Lactobacillus* (LAB), which are beneficial bacteria that normally dominate the vaginal microbiota. Restoration of vaginal microbiota with lactobacilli may protect against and treat bacterial vaginosis and GUTIs [8, 9]. Short-chain fructo-oligosaccharides (scFOSs) and other prebiotics are used to selectively stimulate the growth and activity of lactobacilli and bifidobacteria in the colon [10]. The effects of oral supplementation with galacto-oligosaccharide (GOS) and/or FOS on the gut microbiota were investigated in pregnant women to understand whether the prebiotic effect is transferred to offspring. A study of GOS and FOS supplementation demonstrated a bifidogenic effect on the gut microbiota of pregnant German women; however, no transfer of this effect to their infants was found [11]. There was another study to investigate the effect of maternal FOS ingestion (4 g, twice daily) on the number of bifidobacteria in the maternal and neonatal gut in a randomized, double-blind, placebo-controlled study [12]. This study further confirmed the increased number of bifidobacteria in stools from pregnant women but not in those from neonates by FOS ingestion. These two studies did not aim to evaluate the effect on genitourinary tract infections and birth outcomes, and the period of ingestion was relatively short, i.e., after 25 gestational weeks.

In a review article of the studies of probiotic and prebiotic effects on female urogenital infections, it was concluded that prebiotics should be consumed daily to ensure a continuous effect [13]. Favorable changes in the intestinal microbiota were observed at doses of 4–20 g/d of inulin and/or FOS [14, 15]. However, there is limited evidence on whether maternal oral supplementation with prebiotics affects the vaginal microbiota or birth outcomes. The objective of the present study was to examine whether FOS supplementation (6 g/d, once daily) in women starting in early pregnancy protects them from genitourinary tract infections and improves birth outcomes.

## Materials and methods

### Study design and setting

This study was a randomized, double-blinded, placebo-controlled community-based clinical trial in which women in early pregnancy (6–12 weeks of gestation) were enrolled. The study was conducted in a peri-urban community, *Nandipara,* near Dhaka, Bangladesh, where the International Centre for Diarrhoeal Disease Research, Bangladesh (icddr,b), has been performing clinical studies since 1995 [16]. Nandipara is located 8 km east of the icddr,b campus in Dhaka. Study participants were enrolled over a 2-year period from June 2015 to May 2017. Community health workers (CHWs) performing monthly home visits identified women from the local community who had missed menstrual periods. Pregnancy was confirmed through a standard urine test (Gravindex; Ortho Diagnostics Inc., Raritan, NJ) conducted on a morning urine sample by a trained CHW. After confirmation of pregnancy, the CHWs referred the women to the study physician, who determined the women’s eligibility for the study.

### Participant eligibility

The study physician screened the women on the basis of the study inclusion and exclusion criteria. Pregnant women aged 18–35 years were invited to participate in the study. Women with a history of gestational diabetes or preeclamptic toxemia; any systematic disorders or chronic illnesses; or major gynecological problems or treatments, including myomectomy, knife cone biopsy, uterine or vaginal abnormalities, or three or more previous consecutive spontaneous abortions were excluded from the study. Furthermore, women who had anemia (hemoglobin (Hb) level < 7 gm/dL) and women who took antibiotics within 3 weeks prior to the study were excluded. The participants were further screened for complications in previous pregnancies (stillbirth, preterm labor, complicated instrumental delivery, retained placenta, 3°/4° perineal tear, transverse fetal position, placental abruption, or previous baby weighing < 2.5 kg or > 4.5 kg) by reviewing previous delivery records. The presence of urinary tract infection (UTI) or bacteriuria in a morning midstream fresh urine sample, the presence of abnormal vaginal flora (Nugent score ≥ 7), and a history of irregular bleeding due to an injectable contraceptive (medroxyprogesterone acetate) were examined at screenings. Women with any of these factors were also excluded from study participation.

### Baseline data collection

The study objectives and procedure were explained to the women and her caregiver, and written informed consent was subsequently obtained for enrollment in the study. Relevant information on risk factors for LBW, including demographics, socioeconomic status, and detailed gynecological and obstetrical history, was obtained by interviewing the women. Anthropometric measurements, including height and weight, were obtained, and body mass index (BMI) was calculated. At screening, blood, urine, and stool samples and vaginal swabs were taken. The Hb level, random blood glucose level, blood group, and venereal disease research laboratory (VDRL) were analyzed. A midstream urine sample was collected for routine microscopic examination and urine culture. For the culture of LAB and *Bifidobacterium,* a fresh stool sample was collected and immediately transferred to the icddr,b laboratory in a cold box. A vaginal swab was collected by a female physician to detect abnormal vaginal flora and to determine the Nugent score, a Gram stain scoring system used in the diagnosis of BV [17].

### Interventions

Overall, 300 pregnant women from the community were enrolled. A total of 234 women at 12 weeks (3 months) of pregnancy were screened. Among the 234 screened women, 210 were finally included in the study and randomized to either a FOS group, who received 6 g/day of fructo-oligosaccharide (Meioligo-P Powder®, Meiji Food Materia Co., Ltd., Tokyo, Japan) dissolved in 100 ml of Pocari Sweat® (an isotonic sports drink, Otsuka Pharmaceutical, Japan; ingredients are water, sugar, citric acid, trisodium citrate, sodium chloride, potassium chloride, calcium lactate, magnesium carbonate, and flavoring; energy = 55 kcal, protein = 0 g, fat = 0 g, carbohydrates = 13.3 g, sodium chloride (salt) equivalent = 0.24 g, potassium = 36 mg, calcium = 4 mg, and magnesium = 1 mg), or placebo (100 ml of Pocari Sweat® without FOS), also taken once daily for 6 months (i.e., up to childbirth).

### Randomization and blinding

A random permuted block design (to equalize the number of women in the two groups) was used for the allocation of women to the study groups. All women qualifying for study inclusion were assigned a sequential randomization number that had been designated for either of the groups before the randomization process.

Two randomization tables (one for the FOS group and one for the placebo group) with group codes were generated by an independent researcher (senior scientist). Once generated, the allocation code was given to the research supervisor by the independent researcher in an individual concealed envelope, thereby linking each participant to a randomly assigned group via allocation concealment. Thus, once informed consent had been obtained and the participant had been enrolled in the trial, the next available number from the list was assigned to the participant. The linkage was also manually documented in a spreadsheet (enrollment log), and the participant ID was written on the assigned envelope. Neither the participants nor the researchers, caregivers, or laboratory personnel were aware of the group until all the data analyses were completed and until after the blind review meeting.

### Preparation and administration of the solutions

All solutions were prepared in the field office by an independent research supervisor according to the randomization chart. The appearance of the supplements after dissolving 6 g of FOS in 100 ml of Pocari Sweat® and Pocari Sweat® itself as a placebo was identical. Both solutions were distributed (equal amount, 100 ml) in a bottle for each participant.

The drink was orally administered to the enrolled women at their homes at approximately 8:30 AM for 6 consecutive days per week for 6 months. Administration was supervised by a health worker and a research assistant. The bottle was then rinsed with an additional 10 ml of water, which the participant drank.

### Study procedures during pregnancy and the follow-up of infants

**Fig. 1:**
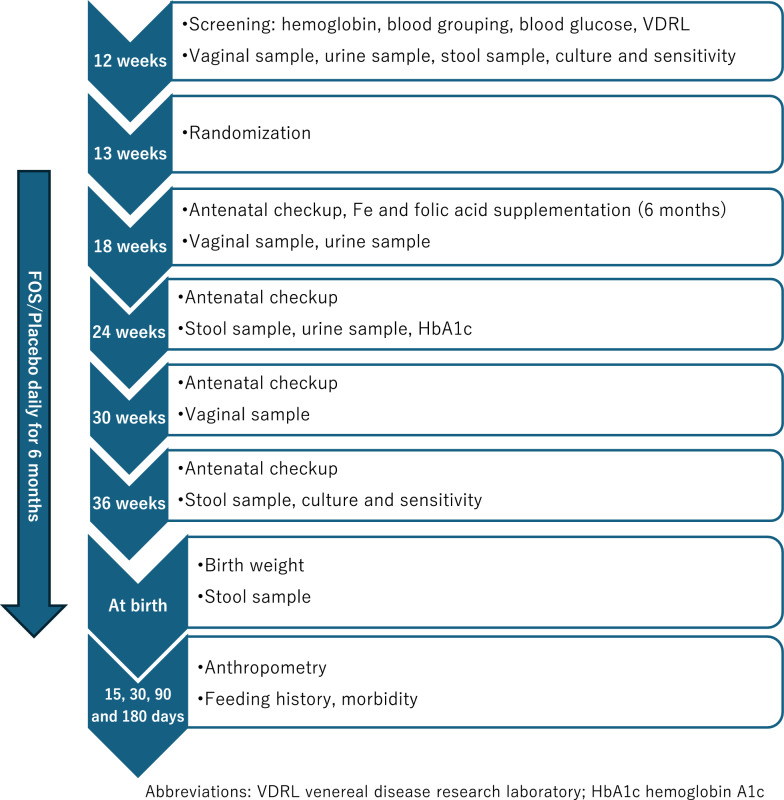
Procedures of the study

The study procedures with specific timings of measurements and sample collection are illustrated in Fig. 1. Routine antenatal check-ups were performed at 6-week intervals at 18, 24, 30, and 36 weeks. To identify gestational diabetes mellitus, HbA1c was evaluated at 24 weeks of gestational age. All HbA1c values were below 6.4%, and no difference was observed between the FOS and placebo groups (Supplemental Table 1).

**Table 1:** General characteristics of pregnant women at enrollment (*n* = 210)

Characteristics	FOS group, n (%) (*n* = 105)	Placebo group, n (%) (*n* = 105)	*p* value^a^
Age, years, mean ± SD	25.0 ± 4	24.0 ± 4.5	0.077
Weight, kg, mean ± SD	46.3 ± 7.6	47.1 ± 8.6	0.505
Height, cm, mean ± SD	149.0 ± 5.9	149.5 ± 5.3	0.551
BMI, mean ± SD	21.1 ± 3.3	21.0 ± 3.3	0.735
Education			0.840
Primary	65 (61.9)	61 (58.1)	
Secondary	34 (32.4)	38 (36.2)	
Above secondary	6 (5.7)	6 (5.7)	
Family income < 9000 BDT	58 (55.2)	62 (59.1)	0.577
RBG, mmol/l, mean ± SD	4.7 ± 0.59	4.7 ± 0.52	0.798

*FOS* fructo-oligosaccharide, *SD* standard deviation, *BDT* Bangladesh Taka, *RBG* random blood glucose

^a^*p* values were calculated via t tests for continuous variables and Chi-square test for categorical variables

A low vaginal specimen was obtained by an appropriately trained female medical person using flocked swabs (COPAN Innovation, Brescia, Italy) for Gram staining. The Nugent score was calculated by assessing the presence of large gram-positive rods (*Lactobacillus* morphotypes; scored from 0 to 4), small Gram-variable rods (*Gardnerella vaginalis* morphotypes; scored from 0 to 4), and curved Gram-variable rods (*Mobiluncus* spp. morphotypes; scored from 0 to 2).

Vaginal swabs obtained at screening (12 weeks) and at 18 and 30 weeks of gestation were tested for *Candida* infection via culture at the icddr,b laboratory. A midstream urine sample was also obtained at 18, 24, and 36 weeks for routine examination. These samples were cultured only when > 6 white blood cells/high-power field (HPF) were observed.

Birth weight was measured, and stool samples were taken at birth. Anthropometry, feeding history, and morbidity were evaluated at Days 15, 30, 90, and 180 after birth.

### Laboratory procedures

#### DNA extraction

The stool samples collected were stored at − 70 °C. For DNA extraction, frozen samples were thawed, and approximately 0.2 g of stool was used according to the instructions of the QIAamp DNA Stool Mini Kit (Qiagen). The bacterial cells present in the stool samples were lysed with lysis buffer. The PCR inhibitors present in the stool samples were then separated from the DNA by using Inhibit EX buffer. The sample was pelleted via centrifugation, and the DNA in the supernatant was purified in QIAamp Mini Spin Columns. The eluted DNA (approximately 200 µl) was stored at − 20 °C.

#### Primer and probe design

To design primers and probes to detect *Bifidobacterium* spp. and *Lactobacillus* spp., 16S rRNA gene sequences were retrieved from the Entrez database of the National Center for Biotechnology Information (NCBI) [18]. The primers and probes used were manufactured by AIT Biotech PTE LTD, Singapore (group-specific primers based on the 16S rDNA sequence are shown in Supplemental Table 2).

**Table 2:** Weight gain of pregnant women over different gestational periods (*n* = 210)

Gestational periods	*n*	FOS group mean ± SD (*n* = 105)	*n*	Placebo group mean ± SD (*n* = 105)	*p* value^a^
Weeks 13–18	91	1.7 ± 2.0	96	1.9 ± 2.3	0.207
Weeks 18–24	86	2.7 ± 2.1	92	2.8 ± 2.7	0.403
Weeks 24–30	82	2.4 ± 2.4	85	3.2 ± 3.8	0.169
Weeks 30–36	42	1.9 ± 3.1	45	2.4 ± 4.3	0.404
Total weight gain between 13 and 36 weeks of gestation	42	8.7 ± 3.4	45	10.0 ± 4.2	0.149

*FOS* fructo-oligosaccharide

^a^*p* values were determined by *t* test

#### Real-time PCR

The total volume of the 25 ml amplification reaction consisted of Platinum™ Quantitative PCR SuperMix-UDG w/ROX (Thermo Fisher Scientific), both primers (each at a 200 nM concentration), a 200 nM TaqMan BHQ probe (AIT Biotech) and purified target DNA. An ABI 7500 Fast Dx Real-Time system (Applied Biosystems) was used for amplification (2 min at 50 °C, 10 min at 95 °C, followed by 45 cycles of 15 s at 95 °C and 1 min at 60 °C) and detection. A no-template control, a negative control, and a positive control were included in each run.

### Outcomes

The primary outcome was the percentage of LBW neonates, and the secondary outcomes were the rates of vaginal and intestinal colonization with LAB and GUTIs at various times of gestation as well as LAB colonization at birth for newborns. The increase in the z score of anthropometric measurements in infants from birth was also examined.

#### LBW

Birth weights were measured at birth. Using the cut-off of 2,500 g, this variable was categorized into two categories: LBW (< 2,500 g) and normal weight. The percentage of LBW were compared between the FOS and placebo group.

#### Bacterial vaginosis

The Nugent score ranges from 0 to 10. A score of 7 to 10 is consistent with bacterial vaginosis [17]. The binary category was made for BV using this cut-off; 0–6 (normal) and 7–10 (BV). Since BV cases are screened out at enrollment, BV incidence was compared between the FOS and placebo group.

#### LAB and Bifidobacterium in the stool of mothers during pregnancy and infants at birth

LAB and *Bifidobacterium* in the stool of mothers at gestational weeks 24 and 36 and in the stool of infants at birth were examined and categorized into “presence” and “absence”. The rates of LBW or *Bifidobacterium* were compared between the FOS and placebo group.

### Statistical analysis

The data are presented as the means and standard deviations. The significance level threshold was set at a *p* value < 0.05. Variables used in the analysis were examined for normality in skewness and kurtosis and confirmed normal distribution. Therefore, for comparisons of categorical variables, the Chi-square test was used, and continuous variables were compared with Student’s t test. Generalized linear models with log-binomial functions were used with covariates. The variables that are known to relate genitourinary tract infections and birth outcomes such as maternal age (continuous), early pregnancy BMI (continuous) and mode of delivery (cesarean section) were considered in the analyses. Statistical analysis was performed using SPSS ver. 29 (IBM Corporation, USA).

### Sample size

The sample size was estimated on the basis of (i) the rates of LAB/*Bifidobacterium* colonization in the gut or vagina; (ii) the rates of UTIs and/or BV; and (iii) the rate of LBW. The estimated percentages of LAB or Bifidobacterium colonization [11], GUTIs [5] and LBW [19] in the population are 25%, 30%, and 35%, respectively. Sample size for comparing two proportions was used [20]. By considering clinical significance, there would be a 20% difference in each outcome between the FOS and placebo groups. The sample size per group was calculated as 59, 72, and 82 for LAB or *Bifidobacterium* colonization, GUTIs, and LBW evaluation, respectively, with a significance level of 0.05 and a power of 0.80. Therefore, accounting for a 5% dropout rate, 105 participants were included in each group (a total of 210 participants).

## Results

**Fig. 2:**
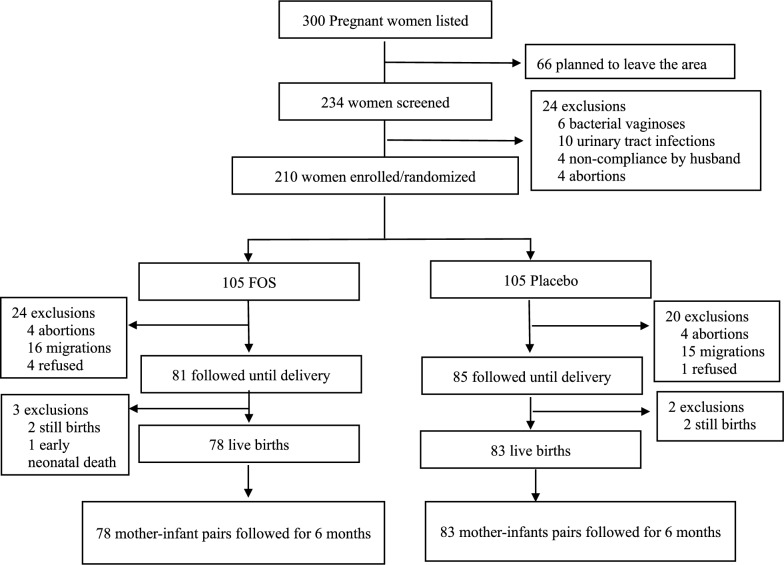
Flowchart of the study participants

Figure 2 shows the flowchart of the study participants. Overall, 300 pregnant women were contacted, 66 of whom migrated outside the study area and could not be located. They seem to have moved out to other family places for delivery. The remaining 234 pregnant women were screened, 24 of whom were excluded. Finally, 210 participants were included in the study, and 105 received the fructo-oligosaccharide (FOS) plus Pocari Sweat®, and 105 received placebo (Pocari Sweat® alone). After inclusion, 44 mothers were lost to follow-up. Among the remaining 166 mothers, stillbirth and early neonatal death occurred for 5 participants. The remaining 161 mother–infant pairs were followed up for 6 months.

Table 1 shows general characteristics of pregnant women at enrollment. The treatment and control groups did not differ with respect to age, weight, height, BMI, education level, or family income at the time of randomization.

Table 2 shows the weight gain of pregnant women. The rates of weight gain over different gestational periods were comparable between the mothers receiving FOS and those receiving placebo. The mean total weight gain between 13 and 36 weeks of gestation was 8.7 kg for mothers receiving FOS and 10.0 kg for those receiving placebo; the difference was not statistically significant.

### Birth events and birth weight

Among the 161 women, 116 (72.0%) had a normal vaginal delivery (NVD), and 45 (28.0%) had delivery via cesarean section. Among the newborns, birth weight was recorded for 159 participants (159/161; 98.8%). Overall, the mean birth weight of the newborns was 2.8 ± 0.4 kg, and the mean birth length was 47.3 ± 2.5 cm. Table 3 shows anthropometric measurements at births. There were no differences in the mean birth weight, length, or mid-upper arm circumference (MUAC) of the babies born to the mothers receiving FOS or placebo. Low birth weight was observed with a lower frequency in the FOS group than in the placebo group with no statistical difference (15.6% vs. 19.5%, *p* = 0.650). Further analyses were conducted by controlling factors known to affect birth weight, such as gestational week at birth and maternal early pregnancy BMI. After controlling for these factors, no difference was observed between the groups (adjusted risk ratio (RR) (95% CI) = 0.86 (0.43–1.70), *p* = 0.654). Further adjustments with other factors such as gravida, sanitation, and family income did not improve the goodness of fit in the Bayesian Information Criterion (BIC).

**Table 3:** Anthropometric measurements at birth (*n* = 159)

Parameters	FOS group, mean ± SD, (*n* = 77)	Placebo group, mean ± SD (*n* = 82)	*p* value^a^
Weight, kg	2.8 ± 0.3	2.8 ± 0.4	0.671
Length, cm	47.2 ± 2.8	47.2 ± 2.2^b^	0.809
MUAC, cm	9.4 ± 0.6	9.5 ± 0.8^b^	0.182
Low birth weight, n (%)	12 (15.6)	16 (19.5)	0.650

*FOS* fructo-oligosaccharide, *SD* standard deviation, *MUAC* mid upper arm circumference

^a^*p* values were determined via a t test for continuous variable and Chi-square test for categorical variable

^b^In the placebo group, there was one missing value for both length and MUAC

### Changes in anthropometric measurements over 6 months after birth

The number of infants who completed the 6-month postnatal follow-up was 60 (60/77, 77.9% of live births) in the FOS group and 67 (67/84, 79.8% of live births) in the placebo group. Almost all the infants (126/127, 99.2%) were breastfed after birth. Infant body weight gain from birth to 6 months of age was 4.36 kg in the FOS group and 4.19 kg in the placebo group; the difference was not statistically significant (*p* = 0.246).

#### *Lactobacillus* and *Bifidobacterium* in the stool of mothers and infants

At enrollment, 75.3% (64/85) and 67.7% (63/93) of mothers in the FOS and placebo groups, respectively, had LAB in their stool cultures. For *Bifidobacterium*, 64.7% (55/85) and 66.7% (62/93) of mothers in the FOS and placebo groups were positive, respectively, at enrollment. There was no significant difference in the rates of LAB and *Bifidobacterium* positivity at enrollment (*p* = 0.266). At week 24, the rates of LAB colonization increased to 89.5% (77/86) in mothers in the FOS group and 90.2% (83/92) in mothers in the placebo group. In contrast, the rates of *Bifidobacterium* positivity decreased to 51.2% (44/86) and 43.5% (40/92) in mothers in the FOS and placebo groups, respectively. At week 24, the rates of LAB and *Bifidobacterium* were not significantly different between mothers in the FOS group and mothers in the placebo groups (*p* = 0.880 and *p* = 0.305, respectively). At week 36, although there were smaller numbers of stool samples collected (51.6%, 83/161 of births), 97.6% (41/42) and 97.7% (43/44) of mothers in the FOS and placebo groups, respectively, were positive for LAB, whereas 88.1% (37/42) and 77.3% (34/44) in the respective groups were positive for *Bifidobacterium*. There was no significant difference between the groups in terms of LAB or *Bifidobacterium* positivity (*p* = 0.973 and *p* = 0.186, respectively).

### Lactobacillus and Bifidobacterium in stools of infants

At birth, the LAB colonization rate was higher in infants in the FOS group than in infants in the placebo group (68.8%, 53/77 in FOS and 51.2%, 42/82 in placebo, *p* = 0.024). Table 4 shows the results of LAB rates in stools of infants between the groups. Compared with infants in the placebo group, infants in the FOS group showed a higher rate for LAB (RR (95% CI) = 1.34 (1.04–1.74), *p* = 0.025). In the multivariable model, after adjusting for maternal age and LAB presence at enrollment, the rate of *Lactobacillus* remained greater in the FOS group than in the placebo group (adjusted RR (95% CI) = 1.45 (1.12–1.88), *p* = 0.005). Further adding other factors such as gravida, sanitation, and family income did not improve the goodness of fit in BIC. After the exclusion of infants delivered via cesarean section, the LAB colonization rate remained significantly greater in the FOS group than in the placebo group. The *Bifidobacterium* colonization rate did not differ between the groups.

**Table 4:** LAB in infant stool at birth (*n* = 159)

Intervention group	Crude model (*n* = 159)		Adjusted model (*n* = 137)^a^	
	RR (95% CI)	*p* value	RR (95% CI)	*p* value
FOS	1.34 (1.04–1.74)	0.025	1.45 (1.12–1.88)	0.005
Placebo	Ref		Ref	

*FOS* fructo-oligosaccharide, *RR* risk ratio, *CI* confidence interval

^a^Adjusted for mother’s age and LAB colonization at enrollment, missing values for LAB colonization at enrollment, *n* = 22

### Vaginal microbiology

BV was observed in 4.3% (4/89) and 3.1% (3/94) of women in the FOS and placebo groups, respectively. BV incidence during pregnancy did not differ between the groups (*p* = 0.716).

Ruling out the effect of vaginal candidiasis on birth outcome was also attempted in this study. *Candida* spp. were isolated from the vaginal swabs obtained at enrollment in 16.5% (15/91) of the women*.* The rate of *Candida* spp. isolation from the vaginal swabs obtained at week 30 was higher in women in the FOS group than in women in the placebo group (42.3% vs. 24.1%, *p* = 0.043). However, this difference did not persist after adjusting for *Candida* spp. positivity at enrollment (*p* = 0.091).

### Urine microbiology

At enrollment, half of the women (111/206, 53.9%) had no bacteria in their urine cultured on blood agar or MacConkey agar, and microscopic assessment with Gram staining was performed. The remaining women (*n* = 95) presented clinically nonsignificant infections, such as *Staphylococcus* and *E. coli,* with < 1 × 10^4^ CFU/ml.

When a cut-off of > 6 white blood cells/HPF was used, the rates of UTI were 36.6% (34/93) and 34.0% (33/97) in the FOS and placebo groups, respectively. The rates of UTIs during pregnancy were not different between the FOS and placebo groups (*p* = 0.714).

## Discussion

This community-based, double-blind, randomized controlled trial was performed to evaluate whether prebiotic (FOS) supplementation during pregnancy reduced the incidence of GUTIs and improved pregnancy outcomes. There was no difference between the FOS and placebo groups with respect to birth weight. The rate of LAB colonization in stool samples obtained at birth was significantly greater in infants of mothers who received FOS than in those whose mothers received placebo.

We observed that the mean birth weight was 2.8 kg and the mean birth length was 47.2 cm in babies of mothers receiving FOS or placebo. Both values were comparable to national data on birth weight and length [16]. The percentage of LBW newborns for mothers receiving FOS was lower than that for mothers receiving placebo (15.6% vs. 19.5%). However, this difference was not statistically significant, even after controlling factors known to affect birth weight, such as gestational age at birth and maternal early pregnancy BMI. Both LBW values were lower than the national value of 22.6% [16]. The percentage of stillbirths in this study was 1.9%, which was also lower than that reported in Bangladesh (2.8%) [21]. Since the number of stillborn infants was small, this study was limited in its ability to assess the differences between groups. Previous studies conducted in Bangladesh identified maternal education and chronic comorbidities including hypertension and diabetes as risk factors for stillbirth [21, 22]. This study excluded women with a history of high-risk pregnancy such as hypertension and other chronic diseases. This might be the reason for this low rate of stillbirths.

We found a significantly higher rate of LAB colonization (typically detected through sensitive polymerase chain reaction (PCR) methods) in stool samples from newborns whose mothers received FOS than in those whose mothers received placebo. These findings support observations that FOS consumption increases the relative abundance of operational taxonomic units belonging to *Lactobacillus* [23]. However, the impact of the outgrowth of these beneficial bacteria on the growth of children could not be established during the first 6 months after birth, as no between-group differences in anthropometric parameters were observed. However, the mean weight at 6 months in both groups combined was 7.1 (SD: 0.86) kg, which is comparable with the 50th percentile reference value (7.3 kg for girls and 7.9 kg for boys) of the World Health Organization [24]. Long-term follow-up studies with larger sample sizes are needed to examine whether LAB colonization early in life is mediated through FOS supplementation.

A cohort study conducted in the USA reported 8.1% as the incidence of UTIs during pregnancy. There is no study found on the incidence of UTIs during pregnancy in other countries. Since the nature of the limited duration of pregnancy with multiple antenatal clinic visits, the incidence during the pregnancy might be similar to the period prevalence. The prevalence of UTIs during pregnancy in rural Bangladesh was reported as 9% [25]. In another study conducted in Bangladesh, the prevalence of UTIs was 8.6% [26]. A cohort study of antenatal clinic attenders in Africa revealed that up to 40% of pregnant women have trichomoniasis and bacterial vaginosis [27]. Although the UTI rates were comparable to those reported in these studies, the BV rate was much lower in both the FOS and placebo groups (4.3% and 3.1%, respectively). Information on BV rates among pregnant women in low-income countries is limited. Owing to the limited number of cases of BV, the hypothesis that prebiotic administration would prevent BV could not be addressed.

Interestingly, we observed a mean gestational weight gain irrespective of group (8.7 and 10.1 kg in the FOS and placebo groups, respectively) was greater than that reported for pregnant women in Bangladesh (6.5 kg [28] or 6.0 kg [29]). Although it might be explained that there was an effect from previous studies conducted in the study area, the greater weight gain in our study might have been attributed to the intake of an isotonic solution (Pocari Sweat®), which was used to dissolve the FOS or was given alone as a placebo. It is likely that the energy and electrolytes obtained by consuming Pocari Sweat® directly or indirectly affected weight gain, which masked any additional (small) FOS prebiotic effect attained in our study. Mothers in both study groups showed optimal weight gain, which might have offset the difference in the percentage of LBW infants between the FOS and placebo groups [30]. Nevertheless, these findings may have public health implications for achieving optimal gestational weight gain, as low pregnancy weight gain is common in Bangladesh [31]. Further studies are needed to examine the independent effect of the isotonic solution on gestational weight gain.

One of the strengths of the study is that pregnant women in early gestation, i.e., 6–12 weeks, were identified. This strength was possible through community surveillance via monthly home visits in the study area. It was important to have 6 months of FOS/placebo supplementation, and it was needed to randomize at 13 weeks of gestation. Moreover, freshly prepared solutions at the community field office were delivered to participants at home, and their intake was directly observed by the research staff. Therefore, high rates of compliance with the intervention products were achieved. 

There were several limitations of the study. First, by excluding women with previous LBW pregnancy, bacteriuria, and BV, the risk of LBW may have been reduced in the present study, thereby reducing the statistical power to detect effect differences [32]. The number of BV cases observed during pregnancy was small in both groups. Second, while designing the study, we expected an LBW rate of 35%, with an observed difference from the placebo of 20% and the power of 0.80. However, we observed only a 4% difference. The power with the actual effect size found in the study was only 0.12. Third, a dropout rate of 5% was used to calculate sample size. However, 23% of women were dropped during the follow-up. This may have limited the statistical power to detect the observed effect. Fourth, the PCR results for LAB or *Bifidobacterium* were presented as “present” or “absent”. Studies with quantitative assessments are warranted to confirm these findings [12].

## Conclusion

A prebiotic FOS supplement did not affect birth outcomes in pregnant women in Bangladesh. However, this prebiotic supplement had a beneficial effect on the outgrowth of *Lactobacillus* in the stool of infants of mothers who received the FOS supplement. Further studies are needed to confirm the effect of FOS supplementation on pregnant women with a large sample size.

## Supplementary Information

Additional file 1.

Additional file 2.

## Data Availability

The datasets used and analyzed during the current study are available from the corresponding author upon reasonable request.

## Abbreviations

BIC: Bayesian information criterion
BDT: Bangladesh Taka
BMI: Body mass index
CHWs: Community health workers
CI: Confidence interval
FOS: Fructo-oligosaccharide
GOS: Galacto-oligosaccharide
GUTIs: Genitourinary tract infections
HbA1c: Hemoglobin A1c
HPF: High-power field
LAB: Lactic acid bacteria,* Lactobacillus*
LBW: Low birth weight
MUAC: Mid-upper arm circumference
NCBI: National Center for Biotechnology Information
RR: Risk ratio
PCR: Polymerase chain reaction
RBG: Random blood glucose
scFOS: Short-chain fructo-oligosaccharide
SD: Standard deviation
UTI: Urinary tract infection
VDRL: Venereal Disease Research Laboratory
