# Supplementary material for: Effects of fructo-oligosaccharides on genitourinary tract infections and birth outcomes in pregnant women: a randomized controlled trial in Bangladesh
Source: Trop Med Health. 2025 Aug 13;53:106. doi: 10.1186/s41182-025-00788-4 (PMC12344985; doi:10.1186/s41182-025-00788-4)
Supplement: Supplementary file 1 — Additional file 1. [file 41182_2025_788_MOESM1_ESM.docx]

Supplemental Table 1. HbA1c at Gestational Week 24 by FOS and Placebo Group

|  | FOS | Placebo | *p* value |
| --- | --- | --- | --- |
| HbA1c (%), means±SD | 4.97±0.59 | 4.96±0.48 | 0.800 |

*P* value was calculated by t test. HbA1c hemoglobin A1c, SD standard deviation.
