# Supplementary material for: Effects of fructo-oligosaccharides on genitourinary tract infections and birth outcomes in pregnant women: a randomized controlled trial in Bangladesh
Source: Trop Med Health. 2025 Aug 13;53:106. doi: 10.1186/s41182-025-00788-4 (PMC12344985; doi:10.1186/s41182-025-00788-4)
Supplement: Supplementary file 2 — Additional file 2. [file 41182_2025_788_MOESM2_ESM.docx]

Supplemental Table 2. Group-specific primers based on 16S rDNA sequences

| Target Bacteria | Primer and Probe | Sequence (5́´˗ 3´) |
| --- | --- | --- |
| *Bifidobacterium* spp. | F-bifido | CGCGTCYGGTGTGAAAG |
|  | R-bifido | CCCCACATCCAGCATCCA |
|  | bifido probe | FAM-AACAGGATTAGATACCC-BHQ-1 |
| *Lactobacillus* spp. | F-lacto | GAGGCAGCAGTAGGGAATCTTC |
|  | R-lacto | GGCCAGTTACTACCTCTATCCTTCTTC |
|  | lacto probe | HEX-ATGGAGCAACGCCGC-BHQ-1 |
